# Mechanism-Driven Evolution of Fertility-Sparing Treatment and Precision Management of Special Populations in Endometrial Cancer: A Review

**DOI:** 10.3390/cancers18172741

**Published:** 2026-08-24

**Authors:** Kai-Bing Qu, Chun-Lin Pan, Ming-Yue Zhang, Zhuo-Ying Du, Sheng-Qian Wang, Shu-Li Yang, Yu-Mei Wu, Jian-Dong Wang, Yue He

**Affiliations:** 1 Department of Gynecologic Oncology, Beijing Obstetrics and Gynecology Hospital, Capital Medical University, Beijing Maternal and Child Health Care Hospital, Beijing 100026, China; qukaibing1112@mail.ccmu.edu.cn (K.-B.Q.);; 2 Clinical Medical Laboratory, Capital Medical University, Beijing 100069, China

**Keywords:** endometrial cancer, fertility-sparing treatment, metabolic abnormalities, molecular classification, progestin resistance

## Abstract

Endometrial cancer is increasingly diagnosed in patients who have not yet completed childbearing. For carefully selected patients with grade 1 endometrioid endometrial carcinoma confined to the endometrium, fertility-sparing treatment (FST) can preserve reproductive potential but requires rigorous histologic surveillance. In eligible patients, oral progestins and/or the levonorgestrel-releasing intrauterine system (LNG-IUS) remain the mainstay of fertility-sparing treatment, with hysteroscopic lesion resection incorporated in selected cases. Obesity, polycystic ovary syndrome, abnormalities in glucose metabolism, molecular subtype, and primary or acquired progestin resistance collectively contribute to heterogeneity in treatment response and the risk of recurrence. This narrative review critically integrates current guidelines, randomized controlled trials, prospective studies, retrospective cohorts, and early exploratory evidence to evaluate the biological rationale, clinical positioning, efficacy, safety, and maturity of evidence for progestin-based therapy, metabolic interventions, combined endocrine approaches, molecularly guided strategies, and exploratory immunotherapeutic approaches. We further propose an integrated clinical pathway encompassing candidate selection, molecular assessment, response evaluation, transition to pregnancy, retreatment after recurrence, and timely conversion to definitive surgery. Importantly, this review distinguishes guideline-supported approaches from adjunctive, investigational, and exploratory strategies. Major evidence gaps include inconsistent definitions of treatment response, limited prospective molecularly stratified data, uncertain reproductive safety of emerging systemic therapies, and insufficient long-term data on pregnancy outcomes and offspring.

## Simple Summary

Endometrial cancer is increasingly affecting individuals who have not yet completed childbearing. For carefully selected patients with early-stage, low-risk disease, fertility-sparing treatment can preserve the uterus while the disease is closely monitored. However, responses to conventional progestin therapy vary, particularly among patients with obesity, polycystic ovary syndrome, abnormal glucose metabolism, Lynch syndrome, mismatch repair-deficient tumors, persistent disease, or recurrent disease. This review describes how fertility-sparing treatment has expanded from oral progestin therapy alone to include local intrauterine therapy, hysteroscopic lesion resection, metabolic interventions, combined endocrine therapy, and selected molecularly guided strategies. It also summarizes the patient populations most likely to benefit from each approach, their limitations, and the circumstances in which fertility-sparing treatment should be discontinued. Integrating pathological, metabolic, molecular, treatment-response, and reproductive factors may help clinicians better balance oncologic safety with the opportunity for future pregnancy.

## 1. Introduction

Although endometrial cancer predominantly occurs in postmenopausal women, the clinical and reproductive needs of patients of childbearing age have become increasingly important. Between 2001 and 2021, the incidence of endometrial cancer among women aged 20–49 years in the United States increased from 86.8 to 113.8 cases per million [1]. Disparities across age, racial, and ethnic groups have also been observed, potentially reflecting differences in metabolic risk factors, sociodemographic characteristics, and access to healthcare [2]. As childbearing is increasingly delayed, a growing number of patients have not completed their reproductive plans at the time of diagnosis, whereas standard definitive surgery results in irreversible loss of fertility.

Against this background, fertility-sparing treatment (FST) has emerged as an important option for selected patients with early-stage, low-risk disease. However, FST should not be regarded as a simple substitute for definitive surgery. Rather, it represents a carefully balanced strategy that preserves the uterus and reproductive potential while accepting the risks of persistent disease, progression, and recurrence. Its fundamental goal is to accommodate patients’ reproductive wishes as much as possible while ensuring that oncologic risks remain within an acceptable and controllable range.

A clear definition of the safety boundaries of fertility-sparing treatment is essential. The joint guidelines of the European Society of Gynaecological Oncology, the European Society of Human Reproduction and Embryology, and the European Society for Gynaecological Endoscopy (ESGO/ESHRE/ESGE) recommend that appropriate candidates should have a strong desire to preserve fertility and fully understand that fertility-sparing treatment is not the standard definitive treatment. The preferred histologic type is grade 1 endometrioid endometrial carcinoma confined to the endometrium, with no evidence of myometrial invasion [3]. The National Comprehensive Cancer Network (NCCN) guidelines similarly emphasize that myometrial invasion and extrauterine disease should be excluded before initiating fertility-sparing treatment and that patients must be able to adhere to long-term, standardized follow-up. Before treatment, the histopathologic diagnosis should be reviewed by an experienced gynecologic pathologist, and magnetic resonance imaging (MRI) or transvaginal ultrasonography should be performed to assess disease extent and exclude myometrial invasion [4].

With the integration of molecular classification into clinical practice, conventional candidate selection based solely on age, histologic type, tumor grade, and imaging findings is no longer sufficient. p53-abnormal tumors, high-grade features, and non-endometrioid histology generally indicate a more aggressive clinical course and should not routinely be considered for fertility-sparing management [3,5,6]. In addition, distinct molecular features, including mismatch repair deficiency/microsatellite instability-high (MMRd/MSI-H), POLE-mutated (POLEmut), and no specific molecular profile (NSMP) tumors, may substantially influence progestin responsiveness, recurrence risk, and subsequent treatment decisions [6]. Accordingly, the clinical question has shifted from simply asking “whether the uterus can be preserved” to determining “how an individualized strategy can be developed, within clearly defined indications, according to each patient’s pathological, metabolic, endocrine, and molecular characteristics.”

The current evidence base encompasses international guidelines, randomized trials, single-arm phase II studies, retrospective cohorts, systematic reviews, and case reports; accordingly, the strength of evidence and clinical relevance vary substantially across treatment strategies. For appropriately selected patients with low-risk disease, oral progestins and the levonorgestrel-releasing intrauterine system (LNG-IUS) are guideline-supported standard approaches. Hysteroscopic lesion resection and metformin-containing regimens may be considered evidence-supported adjunctive strategies in selected clinical settings, whereas gonadotropin-releasing hormone agonist (GnRH-a)-based combination regimens remain individualized investigational options. Glucagon-like peptide-1 (GLP-1)-based therapies, immune checkpoint inhibitors, and estrogen receptor (ER)-targeted agents should currently be regarded as exploratory strategies. Establishing this hierarchy of evidence is intended to avoid interpreting encouraging findings from early-stage studies as routine clinical recommendations [3,4,7].

## 2. Progestin-Based Fertility-Sparing Treatment: From Systemic Therapy to Local Delivery and Local Cytoreduction

### 2.1. High-Dose Oral Progestins: The Endocrine Foundation of Fertility-Sparing Treatment

Fertility-sparing treatment was initially established on the basis of the endocrine-driven pathogenesis of endometrial cancer. The development of endometrioid endometrial carcinoma and atypical endometrial hyperplasia (AEH) is closely associated with prolonged estrogenic stimulation accompanied by insufficient progestogenic counteraction. During the normal menstrual cycle, estrogen primarily promotes proliferation of the endometrial glands, whereas postovulatory progesterone signaling induces the transition of the endometrium from the proliferative to the secretory phase. Through progesterone receptor-mediated signaling, progestins suppress cellular proliferation, promote differentiation, and contribute to the cyclic shedding of the endometrium.

Young patients with low-grade endometrioid endometrial carcinoma frequently have obesity, polycystic ovary syndrome (PCOS), chronic anovulation, or insulin resistance, resulting in prolonged endometrial exposure to estrogen without adequate progestogenic opposition. The American College of Obstetricians and Gynecologists (ACOG) has emphasized that unopposed estrogenic stimulation can promote abnormal endometrial proliferation. Accordingly, the rationale for exogenous high-dose progestin therapy is to restore progestogenic opposition to estrogen-driven proliferation and induce secretory transformation of the lesional glands, decidual-like changes in the stroma, and endometrial atrophy, ultimately promoting regression of the lesion [8,9,10].

Commonly used oral progestins in clinical practice include medroxyprogesterone acetate (MPA) and megestrol acetate (MA). A multicenter phase II study conducted by Ushijima et al. demonstrated that high-dose MPA could induce histologic remission in young patients with endometrial cancer or atypical endometrial hyperplasia (AEH) [11]. The ESGO/ESHRE/ESGE guidelines recommend MA at 160–320 mg/day or MPA at 400–600 mg/day as standard oral regimens [3]. The NCCN guidelines likewise recognize continuous progestin therapy as an important option for patients wishing to preserve the uterus or fertility [4].

Overall, the foundational role of progestin therapy in fertility-sparing treatment has been well established, although durable benefit is not achieved in all patients. A systematic review by Gallos et al. [12] reported a response rate of 76.2%, a recurrence rate of 40.6%, and a live-birth rate of 28.0% among patients with early-stage endometrial cancer undergoing fertility-sparing treatment. Suzuki et al. [13] further highlighted substantial heterogeneity across treatment regimens and reproductive outcomes.

Accordingly, oral progestins remain the cornerstone of fertility-sparing treatment and a first-line option for carefully selected patients with low-risk disease. However, their limitations are equally evident. In patients with a relatively high lesion burden, a prolonged time to complete response, obesity or PCOS-associated metabolic and endocrine abnormalities, an inadequate response to progestin therapy, or recurrent disease undergoing repeat fertility-sparing treatment, progestin monotherapy may be insufficient to simultaneously achieve histologic remission, control recurrence, and maintain durable disease control. These limitations have driven the gradual evolution of fertility-sparing treatment from progestin monotherapy toward more comprehensive strategies incorporating local drug delivery, hysteroscopic cytoreduction, metabolic interventions, and combination therapies.

### 2.2. Levonorgestrel-Releasing Intrauterine System (LNG-IUS): Local Delivery with Reduced Systemic Exposure

Long-term, high-dose oral progestin therapy may cause weight gain, edema, headache, elevated blood pressure, thromboembolic risk, and gastrointestinal discomfort, thereby compromising treatment adherence; these concerns may be particularly relevant in patients with obesity or metabolic comorbidities. In contrast, the levonorgestrel-releasing intrauterine system (LNG-IUS) provides continuous local release of levonorgestrel within the uterine cavity, achieving high progestin concentrations in the endometrium while reducing systemic exposure.

Current guidelines recognize the LNG-IUS as one of the principal progestin-based options for appropriately selected patients. The American College of Obstetricians and Gynecologists (ACOG) states that patients with atypical endometrial hyperplasia (AEH) may receive oral, intrauterine, or combined progestin therapy, with intrauterine administration potentially associated with higher rates of disease regression [8]. The ESGO/ESHRE/ESGE guidelines support the use of a 52 mg LNG-IUS either alone or in combination with oral progestins [3]. Similarly, the NCCN guidelines list the 52 mg LNG-IUS as one of the preferred hormonal approaches for patients who wish to preserve the uterus or fertility [4]. In a prospective phase II study, the overall response rate at 12 months was 83%; subgroup response rates were 90.6% for complex atypical hyperplasia and 66.7% for grade 1 endometrioid endometrial carcinoma, and 9.5% of responders subsequently experienced recurrence [14].

The LNG-IUS is particularly suitable for patients who wish to avoid prolonged exposure to high-dose oral progestins, especially those with obesity or metabolic comorbidities who are concerned about systemic adverse effects. It may also be used as maintenance therapy after complete response or combined with oral progestins to enhance local progestogenic effects. Nevertheless, the LNG-IUS remains fundamentally a progestin-based therapy and therefore cannot fully overcome inadequate progestin responsiveness or a substantial lesion burden. In patients with clearly visible intrauterine lesions, relying solely on medical therapy and waiting for lesion regression may not always be the optimal strategy, providing a clinical rationale for the incorporation of local cytoreduction.

### 2.3. Hysteroscopic Lesion Resection Combined with Progestin Therapy: Uterus-Preserving Local Cytoreduction

Both oral progestins and the LNG-IUS require time to induce a histologic response, whereas conventional dilation and curettage or blind endometrial biopsy may underestimate disease extent and miss higher-grade lesions. In this context, hysteroscopy provides both diagnostic and therapeutic value in fertility-sparing management. Direct visualization allows more accurate assessment of lesion location, extent, and morphology and improves the precision of endometrial sampling. At the same time, resection of localized visible lesions can reduce tumor burden and may create a more favorable local environment for subsequent medical therapy.

Current guidelines have incorporated hysteroscopy into fertility-sparing management. The American College of Obstetricians and Gynecologists (ACOG) considers hysteroscopic examination combined with endometrial sampling to be a more accurate approach for detecting concurrent carcinoma in patients with atypical endometrial hyperplasia (AEH) [8]. The NCCN guidelines state that hysteroscopic evaluation and resection of the tumor may be considered [4]. The ESGO/ESHRE/ESGE guidelines indicate that hysteroscopic resection followed by oral progestins and/or the LNG-IUS may achieve higher complete response and live-birth rates than other approaches [3]. Some comparative studies have also suggested that hysteroscopic resection combined with medical therapy may provide better outcomes than medical therapy alone [15]. A meta-analysis reported a complete response rate of 95.3%, a recurrence rate of 14.1%, and a pregnancy rate of 47.8%; however, most included studies were nonrandomized and showed substantial heterogeneity [3,16].

Nevertheless, the evidence supporting hysteroscopic lesion resection combined with hormonal therapy remains heterogeneous. Most fertility-sparing case series are retrospective or nonrandomized, with substantial differences in patient selection, lesion size and distribution, resection technique, operator experience, adjunctive hormonal regimens, and definitions of complete response. Therefore, these favorable pooled outcomes should not be interpreted as level I evidence [7,15,16]. Theoretically, uterine distension pressure during hysteroscopy may facilitate transtubal dissemination of tumor cells. A 2025 systematic review and meta-analysis found no significant adverse effect of hysteroscopy on the rate of positive peritoneal cytology, disease-free survival, or overall survival; however, heterogeneity across studies was moderate to high, and careful control of intrauterine pressure and procedure duration remains advisable [17].

In the fertility-sparing setting, a “fully resectable lesion” should be clearly demarcated, confined to the uterine cavity, and removable without intentional deep resection into the myometrium, with no imaging evidence of myometrial invasion or extrauterine disease. The procedure should be performed by an experienced hysteroscopist, with efforts to minimize unnecessary intrauterine pressure and injury to the basal endometrium. Excessive resection may increase the risk of intrauterine adhesions and compromise subsequent fertility. Moreover, local treatment cannot correct systemic predispositions such as obesity, PCOS, and insulin resistance. Fertility-sparing management therefore also requires attention to the upstream metabolic and endocrine drivers that may contribute to disease development and recurrence.

## 3. Optimization of Fertility-Sparing Treatment in Special Populations and Clinical Scenarios

As foundational fertility-sparing approaches, including oral progestins, the LNG-IUS, and hysteroscopic lesion resection, have become increasingly established, the central question has evolved from “whether the lesion can be reversed” to “why similar baseline treatments lead to different clinical outcomes.” Such heterogeneity may manifest as differences in the time required to achieve response, persistent disease, recurrence after complete response, or ultimately failure to achieve pregnancy.

The factors underlying this heterogeneity can be broadly understood at three levels. First, metabolic and endocrine conditions, including obesity, polycystic ovary syndrome (PCOS), insulin resistance, and abnormalities in glucose metabolism, may continuously influence estrogen exposure, inflammatory status, and progestin responsiveness. Second, molecular features such as mismatch repair deficiency/microsatellite instability-high (MMRd/MSI-H) and Lynch syndrome may alter treatment sensitivity and recurrence risk. Third, during treatment, patients may develop an inadequate response to progestin therapy, progestin resistance, or recurrence after remission, requiring reassessment of both the potential benefit of continued fertility preservation and its oncologic safety boundaries.

Accordingly, this section discusses special populations and clinical scenarios in the sequence of “metabolic and endocrine background–molecular characteristics–treatment response” and, on this basis, considers when treatment may be optimized or intensified and when the fertility-sparing pathway should be discontinued in a timely manner.

The applicability, advantages, and limitations of the major fertility-sparing strategies are summarized in Table 1.

### 3.1. Patients with Metabolic Abnormalities: Obesity, PCOS, and Abnormal Glucose Metabolism

Young patients with endometrial cancer or atypical endometrial hyperplasia (AEH) frequently present with obesity, polycystic ovary syndrome (PCOS), insulin resistance, impaired glucose tolerance, or diabetes mellitus. Although these metabolic and endocrine abnormalities differ in their clinical manifestations, they often share three major upstream drivers: sustained estrogen exposure, hyperinsulinemia and associated growth-promoting signaling, and chronic low-grade inflammation. Together, these factors may promote persistent endometrial proliferation, reduce progestin responsiveness, and increase the likelihood of delayed remission, recurrence, and adverse reproductive outcomes.

Accordingly, management of patients with metabolic abnormalities should not focus solely on whether intrauterine lesions regress. Body weight, ovulatory function, insulin resistance, and abnormalities in glucose metabolism should also be addressed concurrently. The following sections discuss the distinct mechanisms and clinical management priorities associated with obesity, PCOS, and abnormal glucose metabolism.

**Table 1:** Clinical positioning, suitability, evidence maturity, advantages, and limitations of fertility-sparing strategies for endometrial cancer.

Strategy and Clinical Positioning	Suitable Candidates	Avoid/Use with Caution	Evidence Base and Representative Outcomes	Limitations/Safety
High-dose oral progestins (MPA/MA) [3,4,8,11,12,13] • Guideline-supported first-line FST	• AEH or G1 endometrioid EC confined to the endometrium • Strong fertility desire and reliable follow-up	• Hepatic dysfunction, thrombotic risk, or poor tolerability • No response at 6 months: MDT review; no CR by 12–15 months or progression: transition to definitive treatment	• Guidelines, phase II evidence, and meta-analyses • MA 160–320 mg/day or MPA 400–600 mg/day: response rate 76.2%, recurrence rate 40.6%, and live-birth rate 28.0% [12]	• Weight gain, edema, headache, hypertension, hepatic/gastrointestinal toxicity, or thrombosis • Primary/acquired resistance and recurrence
52 mg LNG-IUS [3,4,8,14] • Guideline-supported first-line FST	• Need to minimize systemic exposure • Obesity/metabolic abnormalities • Combination therapy or maintenance after CR	• Uterine cavity distortion, large fibroids/adenomyosis, or risk of expulsion • Use LNG-IUS monotherapy with caution in patients with a persistent inadequate response after adequate prior progestin therapy	• Guidelines and one prospective phase II study • 12-month response rate 83% (AEH 90.6%; G1 EC 66.7%); recurrence 9.5% [14]	• Bleeding/cramping; malposition/expulsion; rare infection/perforation • Evidence for monotherapy in confirmed EC remains limited
Hysteroscopic resection + progestin [3,4,8,15,16,17] • Evidence-supported adjunctive strategy	• Localized, clearly visible lesion • No myometrial invasion or extrauterine disease • Requires an expert operator	• Diffuse, multifocal, high-grade, or non-endometrioid disease • Need for deep myometrial or extensive basal-layer resection	• Guidelines, comparative studies, and meta-analyses, predominantly nonrandomized studies • CR 95.3%, recurrence 14.1%, pregnancy 47.8% [3,16]	• No level I evidence; • High heterogeneity in technique and patient selection • Bleeding, infection, perforation, adhesions, basal-layer injury, or theoretical risk of dissemination
GnRH-a + aromatase inhibitor or LNG-IUS [3,18] • Individualized investigational strategy	• Obesity, hepatic dysfunction, thrombosis, or progestin intolerance • Persistent estrogenic stimulation with inadequate response	• Low bone mass or severe hypoestrogenic symptoms • Aromatase inhibitor monotherapy is not routine FST	• Retrospective cohorts • CR 93.5% with GnRH-a + LNG-IUS and 95.8% with GnRH-a + letrozole [18]	• Hot flashes, vaginal dryness, arthralgia, or bone loss • Heterogeneous regimens; no validated EC-specific treatment duration
Metformin + progestin [19,20,21,22,23] • Adjunctive/investigational strategy	• Obesity, insulin resistance, diabetes, PCOS, or metabolic syndrome • Use when metabolic optimization is indicated	• Marked renal impairment or poor gastrointestinal tolerability • Not a substitute for antitumor hormonal therapy	• Phase II/follow-up studies, randomized controlled trials, and meta-analyses • MPA + metformin: CR 97%, recurrence 13.1%, 5-year RFS 84.8% [20,21,22,23]	• Gastrointestinal symptoms; long-term vitamin B12 deficiency; rare lactic acidosis • Randomized evidence is inconsistent across subgroups [22,23]
Weight loss, glycemic control, and lifestyle intervention [3,8,24] • Standard supportive management	• Obesity, insulin resistance, diabetes, PCOS, or metabolic syndrome	• Should not be used as a standalone antitumor treatment • Rapid weight loss should be coordinated with pregnancy planning	• Guidelines and observational intervention evidence • Supports metabolic health, pregnancy preparation, and long-term risk reduction [3,8,24]	• Effect on tumor response is indirect and adherence-dependent • Must be coordinated with histologic surveillance and oncologic treatment
GLP-1RA/GIP–GLP-1 dual agonists [25,26] • Exploratory strategy	• Obesity or marked metabolic abnormalities • Prefer standard obesity management or use within a clinical trial	• Should not be used as sole FST for confirmed EC • Avoid during pregnancy	• Current evidence is derived mainly from observational cohorts assessing cancer incidence risk; EC treatment trials are ongoing • May reduce body weight and improve insulin resistance [25,26].	• Efficacy in EC has not been established • No standardized EC-specific dose or treatment duration • Gastrointestinal/gallbladder events
Immune checkpoint inhibitors [6,27,28,29,30] • Early exploratory strategy	• MMRd/MSI-H or Lynch-associated tumors • Investigational setting after inadequate response to conventional treatment	• Not routine first-line FST • Autoimmune disease, pregnancy, or unresolved endocrine/immune toxicity	• Case reports/small case series and ongoing trials • Four CRs and one pregnancy in a Lynch-associated case series [29]	• Efficacy in EC and reproductive safety remain unvalidated • Potential thyroid, pituitary, ovarian, pregnancy, and fetal risks

Abbreviations: AEH, atypical endometrial hyperplasia; CR, complete response; EC, endometrial cancer; FST, fertility-sparing treatment; G1, grade 1; GIP, glucose-dependent insulinotropic polypeptide; GLP-1, glucagon-like peptide-1; GLP-1RA, glucagon-like peptide-1 receptor agonist; GnRH-a, gonadotropin-releasing hormone agonist; LNG-IUS, levonorgestrel-releasing intrauterine system; MA, megestrol acetate; MDT, multidisciplinary team; MMRd, mismatch repair deficiency; MPA, medroxyprogesterone acetate; MSI-H, microsatellite instability-high; PCOS, polycystic ovary syndrome; RFS, recurrence-free survival. The evidence-maturity labels are qualitative categories used in this narrative review for clinical positioning and do not represent formal guideline grading. “Standard” denotes routine approaches supported by guidelines for carefully selected patients; “adjunctive/investigational” denotes strategies supported by emerging but not yet definitive clinical evidence; and “exploratory” denotes approaches for which fertility-sparing efficacy or reproductive safety has not been adequately validated. The Evidence Base column indicates whether support derives primarily from guidelines, randomized trials, prospective phase II studies, retrospective cohorts, meta-analyses, or case-level evidence [3,4,7].

#### 3.1.1. Obesity

The impact of obesity on fertility-sparing treatment can be understood through three interconnected pathways involving estrogen, metabolic signaling, and inflammation. First, increased aromatase activity in adipose tissue enhances peripheral estrogen production, resulting in sustained proliferative stimulation of the endometrium. Second, obesity-associated insulin resistance and hyperinsulinemia may enhance growth-promoting signaling through the insulin/insulin-like growth factor-1 (IGF-1) axis and downstream pathways such as PI3K/AKT/mTOR. Third, a chronic inflammatory milieu characterized by increased interleukin-6 (IL-6), tumor necrosis factor-α (TNF-α), and leptin levels together with reduced adiponectin may further affect estrogen receptor (ER) and progesterone receptor (PR) function through pathways including STAT3 and NF-κB [31,32]. Experimental studies have further suggested that STAT3-mediated upregulation of 24-dehydrocholesterol reductase (DHCR24) may suppress transcription of the progesterone receptor gene (PGR), thereby reducing progestin sensitivity [32]. Collectively, these mechanisms provide a biological rationale for obesity-associated progestin resistance; however, direct causal evidence in human populations remains limited [31,32].

Clinically, obesity or metabolic syndrome has been associated with delayed complete response during fertility-sparing treatment [19,24], suggesting that simply increasing the progestin dose or extending the treatment duration may be insufficient to correct persistent metabolic abnormalities. Accordingly, the therapeutic focus in patients with obesity should shift from “intensifying progestin therapy alone” toward the concurrent implementation of antitumor treatment and weight management. Current guidelines have incorporated weight management into fertility-sparing care. The ESGO/ESHRE/ESGE guidelines emphasize that weight control during treatment may improve the likelihood of response [3], while the ACOG consensus similarly highlights the importance of lifestyle modification and weight management in patients with atypical endometrial hyperplasia (AEH) [8]. Fu et al. reported favorable remission and pregnancy outcomes among overweight patients who achieved weight reduction during fertility-sparing treatment [24]. Therefore, treatment optimization for patients with obesity should include dietary modification, exercise interventions, and structured weight management, with the addition of metabolic pharmacotherapy when appropriate. Once complete response has been achieved, patients should be encouraged to pursue pregnancy or assisted reproductive technology as soon as clinically feasible to minimize the risk of recurrence during a prolonged waiting period.

In recent years, glucagon-like peptide-1 (GLP-1) receptor agonists and dual GLP-1/glucose-dependent insulinotropic polypeptide (GIP) receptor agonists have emerged as potential strategies for fertility-sparing management in patients with obesity. In a study of patients with type 2 diabetes mellitus, Wang et al. reported that the use of GLP-1 receptor agonists (GLP-1RAs) was associated with a reduced risk of several obesity-related cancers, with a similar trend toward a lower risk of endometrial cancer [25]. Dai et al. likewise reported an association between GLP-1RA use and a reduced risk of endometrial cancer among adults with overweight or obesity [26]. However, these findings primarily reflect associations with cancer incidence and do not directly demonstrate a therapeutic effect of GLP-1RAs in patients with established endometrial cancer or atypical endometrial hyperplasia (AEH). Notably, a clinical trial registered on ClinicalTrials.gov (NCT07349641) is evaluating a dual GIP/GLP-1 receptor agonist in combination with the levonorgestrel-releasing intrauterine system (LNG-IUS) in patients with overweight or obesity and AEH or grade 1 endometrial cancer, with the primary aim of improving the pathological complete response rate through weight reduction.

Overall, the role of GLP-1RAs and dual GIP/GLP-1 receptor agonists in fertility-sparing treatment remains exploratory. Their effects on complete response rates, recurrence rates, reproductive safety, and the identification of patients most likely to benefit require validation in prospective studies.

Selected clinical trials evaluating metabolic interventions in fertility-sparing treatment are summarized in Table 2.

#### 3.1.2. Polycystic Ovary Syndrome

Compared with obesity, the impact of polycystic ovary syndrome (PCOS) on fertility-sparing treatment is more prominently characterized by chronic anovulation and the resulting endocrine imbalance. In the absence of ovulation, corpus luteum formation does not occur, resulting in a lack of cyclic progesterone exposure. Consequently, the endometrium remains chronically exposed to a relatively unopposed estrogenic environment and does not undergo regular secretory transformation and shedding.

PCOS is also frequently accompanied by hyperandrogenism and insulin resistance. Hyperinsulinemia can promote ovarian and peripheral androgen production while reducing sex hormone-binding globulin levels, thereby increasing free androgen concentrations and further aggravating endocrine imbalance. Chronic inflammation and alterations in the local endometrial microenvironment may additionally reduce sensitivity to progestin therapy [33]. Therefore, the impact of PCOS on fertility-sparing treatment cannot be attributed simply to obesity or insulin resistance; rather, it reflects the combined effects of ovulatory dysfunction, endocrine imbalance, and metabolic abnormalities. Together, these factors may sustain endometrial proliferation and contribute to delayed complete response, a shorter interval between remission and recurrence, and difficulty achieving pregnancy. Subsequent management should therefore address both oncologic response and reproductive endocrine abnormalities, rather than considering lesion regression as the sole therapeutic objective.

A retrospective study by Wang et al. [34] included 285 patients with atypical endometrial hyperplasia (AEH) or endometrioid endometrial carcinoma who underwent fertility-sparing treatment, of whom 103 had PCOS. Compared with patients without PCOS, those with PCOS had a lower cumulative complete response rate at 16 weeks, required a longer time to achieve complete response, and experienced a shorter interval from remission to recurrence, suggesting that PCOS may adversely affect treatment response and recurrence risk. Harada and Osuga [33] further emphasized that endometrial dysfunction in patients with PCOS cannot be attributed solely to obesity or insulin resistance but may also involve alterations in sex hormone receptors, steroidogenic enzymes, chronic inflammation, and the local endometrial microenvironment.

At present, there is no antitumor regimen specifically designed for patients with endometrial cancer or atypical endometrial hyperplasia (AEH) and concomitant PCOS that is independent of standard fertility-sparing approaches. Treatment therefore remains progestin-based, including oral progestins, the LNG-IUS, or combination therapy. However, compared with the general low-risk population, patients with PCOS require more active concurrent management of metabolic and reproductive endocrine abnormalities, including weight control, improvement of insulin resistance, appropriate use of metformin, management of ovulatory dysfunction, and timely transition to pregnancy after complete response.

For patients planning pregnancy in the near term, conception should be pursued as soon as clinically feasible after complete response, with early referral for assisted reproductive technology when necessary to reduce the risk of recurrence during a prolonged waiting period [3]. For patients without immediate pregnancy plans, maintenance therapy and metabolic management should be strengthened to prevent renewed prolonged exposure of the endometrium to an estrogenic environment without adequate progestogenic opposition.

#### 3.1.3. Impaired Glucose Tolerance, Insulin Resistance, and Diabetes Mellitus

Unlike PCOS, in which chronic anovulation is a prominent feature, the effects of impaired glucose tolerance, insulin resistance, and diabetes mellitus on fertility-sparing treatment are more closely related to hyperinsulinemia and its downstream growth-promoting signaling. Hyperinsulinemia may enhance activation of the insulin-like growth factor-1 (IGF-1) axis and the PI3K/AKT/mTOR pathway, thereby promoting cellular proliferation and inhibiting apoptosis. At the same time, it may reduce sex hormone-binding globulin levels and increase the proportion of free estrogen, further reinforcing estrogen-driven endometrial proliferation [31,32].

Ding et al. [19] reported that metabolic syndrome was an independent risk factor for a prolonged time to complete response among patients with atypical endometrial hyperplasia (AEH) or early-stage endometrial cancer undergoing fertility-sparing treatment. Accordingly, management of patients with abnormalities in glucose metabolism should not focus solely on regression of the intrauterine lesion but should also include glycemic control, improvement of insulin resistance, and comprehensive management of metabolic syndrome.

Metformin is one of the most extensively studied metabolic adjuncts in fertility-sparing treatment. Its potential effects may be mediated through improved insulin sensitivity, reduction in hyperinsulinemia, and modulation of the AMPK/mTOR signaling pathway. In a phase II study of medroxyprogesterone acetate (MPA) combined with metformin, 61 of 63 patients (97%) achieved complete response within 18 months [20,21]. During extended follow-up, 8 of the 61 responders (13.1%) experienced recurrence, with an estimated 5-year recurrence-free survival rate of 84.8% [21]. However, a randomized controlled trial by Yang et al. [22] showed that the benefit of megestrol acetate (MA) combined with metformin over MA alone was not consistent across all patient subgroups. A meta-analysis by Shao et al. [23] suggested that the addition of metformin to progestin therapy may provide an overall benefit; nevertheless, further evidence is required to establish its specific efficacy in patients with endometrial cancer and to clarify long-term outcomes.

Dosing regimens reported in clinical studies should be interpreted as study-specific protocols rather than as universally applicable prescriptions. Mitsuhashi et al. evaluated MPA at 400 mg/day combined with metformin, which was gradually escalated from 750 mg/day to 2250 mg/day [20]. Yang et al., by contrast, compared MA at 160 mg/day alone with MA at 160 mg/day plus metformin 500 mg three times daily [22]. When metformin is considered, renal function, gastrointestinal tolerability, indications for glucose-lowering therapy, concomitant medications, and pregnancy plans should all be taken into account. At present, no standardized metformin dose or treatment duration, independent of the concomitant progestin regimen, has been established as a standard component of fertility-sparing treatment for endometrial cancer.

Taken together, metformin appears to be more appropriately positioned as an adjunctive intervention for patients with obesity, PCOS, insulin resistance, impaired glucose tolerance, or diabetes mellitus, rather than as a standard intensification strategy intended to universally improve fertility-sparing outcomes in all patients.

According to ClinicalTrials.gov, NCT04792749 is a phase III study evaluating the addition of metformin to conventional progestin-based fertility-sparing treatment for early-stage endometrial cancer. Its current recruitment status is listed as “Unknown,” with the last known status being “Recruiting.” NCT04607252 is a phase II/III study evaluating metformin combined with megestrol acetate (MA) in patients with atypical endometrial hyperplasia (AEH), but the study was terminated after enrolling 12 participants. These trial records indicate that the potential roles of metformin in improving treatment response and reducing recurrence remain to be clarified. Further studies are also needed to define the optimal target population, combination regimen, and long-term reproductive safety.

#### 3.1.4. Treatment Intensification in the Context of Metabolic and Endocrine Abnormalities

Beyond foundational management of body weight, glycemic status, and ovulatory function, some patients may continue to experience persistent estrogen-driven stimulation or may be unsuitable for prolonged high-dose oral progestin therapy because of marked obesity, a hypercoagulable state, increased thromboembolic risk, or other tolerability concerns. In these patients, treatment intensification should focus on further reducing estrogenic stimulation and optimizing the endocrine milieu rather than simply increasing progestin exposure.

Accordingly, for patients with marked obesity, intolerance to high-dose progestins, a hypercoagulable state or increased thromboembolic risk, or a history of inadequate response to progestin therapy, gonadotropin-releasing hormone agonist (GnRH-a)-based combinations with an aromatase inhibitor or the LNG-IUS may be considered individualized investigational strategies to reduce estrogenic stimulation or limit systemic progestin exposure. In a retrospective cohort, complete response rates of 93.5% and 95.8% were reported for GnRH-a combined with the LNG-IUS and GnRH-a combined with letrozole, respectively; however, these findings require confirmation in prospective studies [18].

However, the ESGO/ESHRE/ESGE guidelines explicitly state that gonadotropin-releasing hormone analogues should not be used as first-line fertility-sparing treatment [3], and aromatase inhibitors are likewise not currently recommended for routine fertility-sparing management of early-stage disease. Published GnRH-a/aromatase inhibitor-based regimens vary considerably with respect to the specific agents used, dosing intervals, treatment duration, and whether the LNG-IUS is administered concurrently. Consequently, no uniform endometrial cancer-specific dosing regimen or standardized protocol can currently be recommended. These approaches are better regarded as individualized investigational options for carefully selected patients, with active monitoring for hypoestrogenic symptoms, bone loss, and potential reproductive effects.

Although the strategies described above address identifiable metabolic and endocrine drivers, they cannot fully explain the heterogeneity in treatment outcomes among patients. Further consideration of the intrinsic molecular characteristics of the tumor is therefore required.

### 3.2. Molecular Classification-Guided Fertility-Sparing Management: Special Considerations for Lynch Syndrome and MMRd/MSI-H Tumors

Traditional candidate selection for fertility-sparing treatment has relied primarily on age, histologic type, tumor grade, myometrial invasion, and imaging findings. However, advances in molecular classification have shown that even tumors with clinically and pathologically low-risk features may differ substantially in biological behavior and treatment response [6]. Accordingly, the 2025 Chinese expert consensus on molecular classification-guided fertility-sparing management of endometrial cancer recommends molecular testing in patients being considered for fertility preservation, with the aim of identifying high-risk molecular subtypes that may be unsuitable for conservative management and of providing a basis for individualized treatment decisions [6]. This shift marks a transition from candidate selection based solely on clinicopathologic stratification toward an integrated assessment framework incorporating both clinicopathologic and molecular characteristics.

Current evidence on fertility-sparing treatment can be interpreted in the context of the four major molecular subtypes. A 2025 meta-analysis including eight retrospective cohorts and 363 patients reported complete response rates of 66.6%, 48.8%, 50.0%, and 78.4% for POLE-mutated, mismatch repair-deficient (MMRd), p53-abnormal, and no specific molecular profile (NSMP) tumors, respectively, with corresponding recurrence rates of 14.3%, 42.8%, 33.3%, and 18.4% [35]. However, the sample sizes for individual molecular subtypes were small, confidence intervals were wide, and substantial heterogeneity existed across studies in treatment regimens and surveillance strategies. These findings therefore require further validation. Based on the available evidence, the clinical implications of the individual molecular subtypes can be summarized as follows. POLE-mutated endometrial cancer is generally associated with a favorable prognosis; however, its favorable long-term oncologic outcome does not necessarily predict short-term histologic response or reproductive outcomes during fertility-sparing treatment. p53-abnormal tumors retain high-risk biological characteristics and should not be considered routine candidates for fertility-sparing treatment. Although NSMP tumors have shown numerically favorable response rates, this subgroup remains biologically heterogeneous. Particular caution is warranted for MMRd tumors, as several case series have suggested lower rates of response to progestin therapy and a higher risk of recurrence. Overall, molecular classification should be used as a tool to refine candidate selection rather than as a substitute for established clinicopathologic criteria, including low-grade endometrioid histology, disease confined to the endometrium, and the absence of extrauterine spread.

Among the four molecular subtypes, MMRd/MSI-H tumors warrant particular consideration because they may be associated with both a lower response to conventional progestin-based therapy and a higher risk of recurrence, while also representing a potentially actionable subgroup for immunotherapy. Lynch syndrome is primarily associated with germline pathogenic variants in mismatch repair genes. Loss of mismatch repair function leads to the accumulation of DNA replication errors and the development of a microsatellite instability-high (MSI-H) phenotype. These tumors typically exhibit a high mutational burden and increased neoantigen formation, providing a biological rationale for treatment with immune checkpoint inhibitors [6,27]. From the perspective of conventional fertility-sparing treatment, retrospective data summarized in expert consensus statements suggest that patients with MMRd/MSI-H tumors have lower complete response rates and relatively higher recurrence rates than those with NSMP tumors [6]. A meta-analysis by Zhang et al. likewise suggested that patients with MSI-H/MMRd endometrial cancer or atypical endometrial hyperplasia (AEH) have lower overall response rates and an increased risk of recurrence after fertility-sparing treatment [28]. Accordingly, Lynch syndrome-associated or MMRd/MSI-H lesions should not be regarded as equivalent to conventional low-risk endometrioid tumors but rather as a distinct subgroup requiring more cautious and individualized assessment.

At the clinical management level, fertility-sparing treatment in patients with Lynch syndrome or MMRd/MSI-H tumors should be considered cautiously and only when the lesion fulfills all established low-risk criteria, including grade 1 endometrioid endometrial carcinoma or atypical endometrial hyperplasia (AEH), disease confined to the endometrium, no evidence of myometrial invasion or extrauterine disease, and the patient’s ability to adhere to standardized treatment and close surveillance. Before treatment, comprehensive informed consent and multidisciplinary team evaluation are essential [3,6]. The following features should be regarded as exclusion criteria for fertility-sparing treatment: high-grade disease, non-endometrioid histology, p53-abnormal tumors, myometrial invasion, extrauterine spread, disease progression during treatment, or inability to comply with the prescribed follow-up schedule [3,5,6]. In addition, patients with Lynch syndrome require genetic counseling, cascade testing of at-risk family members, and systematic management of the risks of associated malignancies, including colorectal and ovarian cancers. Therefore, fertility-sparing decisions in this population should not focus solely on the choice of a specific treatment regimen but should instead be incorporated into a multidisciplinary management framework involving gynecologic oncology, genetic counseling, reproductive medicine, pathology, and radiology.

Given these immunogenic characteristics, immune checkpoint inhibitors have emerged as a potential exploratory strategy for MMRd/MSI-H or Lynch syndrome-associated lesions. Yang et al. reported four patients with Lynch syndrome-associated endometrial cancer who achieved complete response after fertility-sparing treatment with immune checkpoint inhibitors; one patient subsequently conceived spontaneously and delivered at term, with no recurrence observed during follow-up [29]. These cases provide preliminary evidence supporting the feasibility of incorporating immunotherapy into fertility-sparing management; however, the available evidence remains limited to case-level observations.

However, MMRd/MSI-H status does not necessarily imply that a patient will respond to immunotherapy. Effective immune checkpoint blockade also depends on intact antigen processing and presentation, the infiltration of functional effector T cells into the tumor microenvironment, and multiple immunosuppressive factors, including regulatory T cells, tumor-associated macrophages, myeloid-derived suppressor cells, and immunosuppressive cytokines [27,36,37]. Consequently, substantial heterogeneity in immune phenotypes may exist even within the same molecular subtype. Moreover, no fertility-sparing biomarker panel integrating tumor genotype, antigen-presenting capacity, and characteristics of the immune microenvironment has yet been prospectively validated.

Beyond uncertainty regarding treatment efficacy, reproductive safety represents another major limitation to the incorporation of immunotherapy into fertility-sparing management. Current evidence remains insufficient to adequately address its effects on ovarian reserve, the optimal interval between treatment discontinuation and conception, pregnancy outcomes, and long-term fetal safety. A 2026 systematic review concluded that available clinical data have not demonstrated clear additional gonadotoxicity associated with immune checkpoint inhibitors; however, the evidence remains limited and highly heterogeneous, and menstrual disturbances and ovarian insufficiency have been reported in a small number of observational studies and post-marketing safety reports [30]. Accordingly, immunotherapy should continue to be regarded as an early exploratory strategy for patients with selected molecular characteristics and should preferably be considered within prospective studies or highly individualized multidisciplinary settings rather than as part of routine fertility-sparing treatment. Its use may be considered only in carefully selected patients with an inadequate response to conventional therapy, after comprehensive counseling regarding the uncertainties surrounding reproductive safety and long-term oncologic outcomes.

Clinical trials evaluating immunotherapy in fertility-sparing populations with selected molecular characteristics are summarized in Table 3.

### 3.3. Inadequate Response to Progestin Therapy and Progestin Resistance: Mechanistic Basis and Therapeutic Strategies

“Inadequate response to progestin therapy” and “progestin resistance” are related but not interchangeable concepts. In this review, inadequate response to progestin therapy is used as an overarching clinical term, referring primarily to the absence of the expected histologic improvement or failure to achieve complete response within a predefined assessment interval; it also encompasses patients who show little or no apparent response from the initiation of treatment. By contrast, progestin resistance places greater emphasis on the underlying biological mechanism and refers to the absence of tumor response despite adequate progestin exposure or the progressive loss of responsiveness after an initial response. Importantly, definitions of “nonresponse” and “resistance” remain inconsistent across published fertility-sparing studies, making the reported incidence rates difficult to compare directly between studies.

Mechanistically, the efficacy of progestin therapy depends largely on intact progesterone receptor (PR) signaling. Following binding to PR, progestins promote the transition of the endometrium from a proliferative state toward differentiation and secretory transformation, thereby suppressing disease progression. Reduced PR expression, an altered balance between the PR-A and PR-B isoforms, impaired receptor function, or epigenetic silencing may compromise this signaling pathway, such that an adequate histologic response may not be achieved even with sufficient progestin exposure [38].

However, progestin resistance is unlikely to result from a single receptor abnormality and more often reflects the combined effects of multiple dysregulated signaling pathways. Estrogen receptor (ER) and PR signaling are functionally interconnected. Persistent activation of ER signaling can sustain cellular proliferation and may alter PR expression and function, whereas reduced PR expression or impaired PR activity can weaken the ability of progestins to counteract estrogen-driven proliferation [9,38]. In parallel, PTEN loss and activation of oncogenic pathways such as PI3K/AKT/mTOR and MAPK may allow tumor cells to become partially independent of hormone-dependent signaling. In progestin-resistant endometrial cancer cells, increased PI3K/AKT/mTOR pathway activity has been associated with suppression of autophagy, and mTOR inhibition has been shown to reduce tumor-cell proliferation; however, the supporting evidence remains predominantly preclinical [39].

Metabolic abnormalities may further influence the response to progestin therapy. Obesity, PCOS, insulin resistance, and diabetes mellitus are frequently accompanied by hyperinsulinemia and chronic inflammation, which may not only sustain the activation of proliferative and prosurvival signaling pathways but also alter PR expression or function. For example, the insulin/STAT3/DHCR24/PGR signaling axis suggests that dysregulated insulin signaling may interfere with PR-related pathways, thereby reducing the sensitivity of tumor cells to progestin therapy [32].

Taken together, an inadequate response to progestin therapy does not necessarily indicate insufficient drug exposure. When PR function is impaired, oncogenic signaling pathways remain persistently activated, or metabolic abnormalities coexist, simply increasing the progestin dose may not restore treatment responsiveness. Progestin resistance is therefore better understood as the result of multiple interacting mechanisms, potentially influenced by hormone receptor status, downstream oncogenic signaling, the metabolic and inflammatory milieu, the tumor microenvironment, and treatment-associated clonal evolution or epigenetic alterations. Given this substantial mechanistic heterogeneity, no single biomarker has yet been established that can reliably predict response to progestin therapy and directly guide treatment selection.

For patients with an inadequate response to progestin therapy, the initial step should not be simply to increase the progestin dose or immediately switch treatment regimens, but rather to reassess whether the patient remains an appropriate candidate for fertility-sparing treatment. The histopathologic diagnosis should be reviewed, together with repeat imaging and, when necessary, hysteroscopic reassessment, to exclude high-grade disease, myometrial invasion, extrauterine spread, or residual localized lesions that have not been adequately treated. If the patient continues to meet the criteria for fertility-sparing management after reassessment, treatment may be intensified according to the individual clinical context. Potential approaches include the LNG-IUS combined with oral progestins, hysteroscopic lesion resection followed by medical therapy, or GnRH-a combined with an aromatase inhibitor or the LNG-IUS. In patients with concomitant obesity, PCOS, or insulin resistance, weight and metabolic management should be implemented concurrently [3,15,16,38]. Conversely, if reassessment indicates disease progression, the emergence of high-risk pathologic or molecular features, or an inability to adhere to close surveillance, fertility-sparing treatment should not be prolonged, and timely transition to standard definitive treatment is warranted.

For patients who continue to show an inadequate response or progestin resistance despite adequate progestin therapy, limited retrospective evidence suggests that alternative combination regimens may have a role as salvage fertility-sparing strategies. In a retrospective analysis of 61 patients with progestin-resistant atypical endometrial hyperplasia (AEH) or early-stage endometrial cancer, Chen et al. reported an overall complete response rate of 90.2% with GnRH-a-based repeat fertility-sparing treatment. Among responders, the recurrence rate was 34.5%, and 51.3% of patients who desired pregnancy subsequently achieved pregnancy [40]. However, these findings were derived from a retrospective cohort with heterogeneous treatment regimens and therefore are insufficient to support routine clinical recommendation.

In patients who remain appropriate candidates for fertility-sparing treatment but are unlikely to derive sufficient benefit from conventional treatment intensification, dysregulation of ER/PR signaling provides an additional avenue for exploration. Conventional progestin therapy relies primarily on PR-mediated antiproliferative effects. When ER signaling remains persistently active, PR expression is reduced, or the balance between ER and PR signaling is disrupted, further intensification of progestin therapy alone may not achieve an adequate therapeutic response. Accordingly, ER-targeted therapy should not be viewed simply as a replacement for conventional progestin treatment, but rather as an investigational strategy aimed at suppressing persistent estrogen-driven proliferation and potentially providing an alternative therapeutic approach for patients with an inadequate response to progestin therapy.

Giredestrant is a next-generation oral selective estrogen receptor degrader (SERD) that suppresses estrogen signaling by promoting ER degradation. The ongoing phase II single-arm trial NCT05634499 is evaluating giredestrant monotherapy in patients with grade 1 endometrioid endometrial carcinoma. Eligible patients are required to have grade 1 endometrioid histology, with magnetic resonance imaging (MRI) confirming the absence of deep myometrial invasion and extrauterine disease.

However, ER-targeted therapy remains exploratory in the fertility-sparing setting. Mature data regarding complete response rates, recurrence risk, subsequent pregnancy outcomes, and long-term safety are still lacking; therefore, these approaches cannot currently replace established progestin-based treatment strategies. The discussion above has primarily focused on patients who fail to achieve the expected response during treatment or who progressively lose responsiveness after an initial response. A distinct clinical scenario that requires separate consideration is recurrence after a previously achieved complete response.

### 3.4. Management of Recurrent Disease: Indications for Repeat Fertility-Sparing Treatment and Criteria for Discontinuation

Unlike persistent inadequate response, this section focuses on patients who develop recurrent disease after previously achieving a complete response. Because the uterus remains in situ, recurrence remains a major concern during long-term management. Repeat fertility-sparing treatment after recurrence should be restricted to carefully selected patients who continue to have a strong desire to preserve fertility, whose recurrent lesions remain confined to the uterine cavity, whose histopathologic features continue to meet low-risk criteria, who have no evidence of myometrial invasion or extrauterine disease, and who are able to adhere to close surveillance [3].

Among patients who developed intrauterine recurrence after an initial complete response, Lee et al. reported a response rate of 78% with repeat progestin therapy [41]. These findings suggest that carefully selected patients with intrauterine recurrence may still derive benefit from repeat progestin-based treatment. However, the available evidence is derived primarily from retrospective studies and should not be routinely extrapolated to all patients with recurrent disease.

Repeat fertility-sparing treatment after recurrence should not be regarded as a simple repetition of the initial regimen. At recurrence, at least five dimensions should be reassessed: (1) the recurrence interval and whether the initial complete response was durable or short-lived; (2) the current extent, grade, and histology of the lesion, with exclusion of myometrial invasion or extrauterine disease; (3) the initial treatment regimen, treatment response, time required to achieve complete response, and treatment tolerability; (4) newly available molecular findings and changes in metabolic and endocrine risk factors; and (5) the patient’s subsequent reproductive plans and the feasibility of achieving pregnancy in the near term. Potentially modifiable factors that may contribute to recurrence should also be identified and addressed, including a relatively high initial lesion burden, delayed complete response, inadequate maintenance therapy, persistent obesity, PCOS or insulin resistance, delayed attempts at conception, and a history of inadequate response to progestin therapy.

Based on this reassessment, a risk-stratified approach may be used to guide salvage treatment. For patients whose initial complete response was durable and who subsequently develop a late, intrauterine recurrence that continues to meet low-risk criteria, repeat fertility-sparing treatment may be considered according to the previous regimen and current disease characteristics. Potential options include repeat progestin therapy, the LNG-IUS, hysteroscopic lesion resection combined with medical therapy, GnRH-a-based regimens, and metabolic optimization. By contrast, early recurrence after a prolonged time to initial complete response, multiple recurrences, or the emergence of molecular or pathologic features suggestive of increasingly aggressive disease should prompt stronger consideration of conversion to definitive surgery. These decisions should be made within a multidisciplinary team and should explicitly incorporate the likelihood of achieving pregnancy within a clinically acceptable timeframe.

Repeat fertility-sparing treatment should not be interpreted as a relaxation of established oncologic safety boundaries. Timely transition to standard definitive treatment is warranted in the presence of recurrent disease occurring repeatedly or at short intervals, disease progression, high-grade or non-endometrioid histology, p53-abnormal or other high-risk molecular features, suspected myometrial invasion or extrauterine disease, inability to adhere to surveillance, or when further delay of definitive treatment may compromise oncologic safety. Whether patients are receiving an initial standard regimen, treatment intensification, or repeat fertility-sparing treatment after recurrence, these safety boundaries should be maintained through continuous and standardized reassessment throughout the treatment course.

## 4. Treatment Evaluation, Transition to Pregnancy, and Management After Completion of Childbearing

Whether patients receive standard initial therapy, individualized treatment intensification, or repeat fertility-sparing treatment after recurrence, all strategies should be incorporated into a unified framework of dynamic assessment and reproductive management. Evaluation of fertility-sparing treatment should encompass both oncologic and reproductive outcomes. Oncologic endpoints include the complete response (CR) rate, time to CR, persistent disease, progression, recurrence, and duration of response, whereas reproductive endpoints include pregnancy rate, use of assisted reproductive technology (ART), miscarriage, and live birth. Complete response should be confirmed histologically and should not be inferred solely from symptoms or imaging findings. In long-term assessment, “success” should be understood as a multidimensional outcome that includes achievement of an initial histologic CR, durable disease control without delaying necessary definitive treatment, fulfillment of the patient’s reproductive goals when feasible, and establishment of an appropriate management plan after completion of childbearing. Accordingly, a single negative biopsy or a short-lived CR should not automatically be regarded as long-term treatment success.

The ESGO/ESHRE/ESGE guidelines recommend that complete response should generally be achieved within 6–12 months after initiation of fertility-sparing treatment, with a maximum treatment duration of 15 months. If no response is observed after 6 months of therapy, multidisciplinary discussion is recommended to reassess the risks and benefits of continuing the fertility-sparing pathway [3]. Treatment success should be confirmed by two consecutive negative endometrial biopsies performed at least 3 months apart [3]. The NCCN guidelines similarly recommend endometrial assessment every 3–6 months during treatment and timely transition to standard treatment in the event of nonresponse, persistent disease, recurrence, or progression [4]. However, outcome assessment remains incompletely standardized across published studies. Substantial differences exist in sampling techniques, the number of negative biopsies required to confirm complete response, assessment time points, whether atypical endometrial hyperplasia (AEH) and grade 1 endometrial cancer are analyzed together, definitions of persistent disease and resistance, and recurrence endpoints. This heterogeneity limits direct comparisons of treatment efficacy across different strategies and supports the development of a core outcome set incorporating standardized oncologic and reproductive endpoints [7].

For patients who wish to conceive, complete response should be regarded as a prerequisite for entering the reproductive phase rather than as the final endpoint of fertility-sparing treatment. Reproductive medicine should be integrated early into the treatment pathway rather than introduced only after a prolonged period of infertility. Once complete response has been confirmed, patients may be referred to a reproductive endocrinology and infertility team for assessment of ovulatory function, ovarian reserve, partner-related factors, and the potential need for ovulation induction or assisted reproductive technology (ART). This is particularly important for patients with persistent obesity, PCOS, chronic anovulation, insulin resistance, or other endocrine abnormalities after tumor remission. The ESGO/ESHRE/ESGE guidelines recommend attempting pregnancy as soon as clinically feasible after remission and using ART when appropriate to shorten the interval to conception [3]. If pregnancy is not achieved promptly, prolonged unmonitored waiting should be avoided; reproductive and oncology teams should jointly reassess barriers to conception, maintenance therapy, and surveillance. After completion of childbearing, definitive surgery is recommended because the retained uterus remains a major site of recurrence, typically in the form of total hysterectomy; ovarian preservation or removal should be individualized.

**Figure 1:**
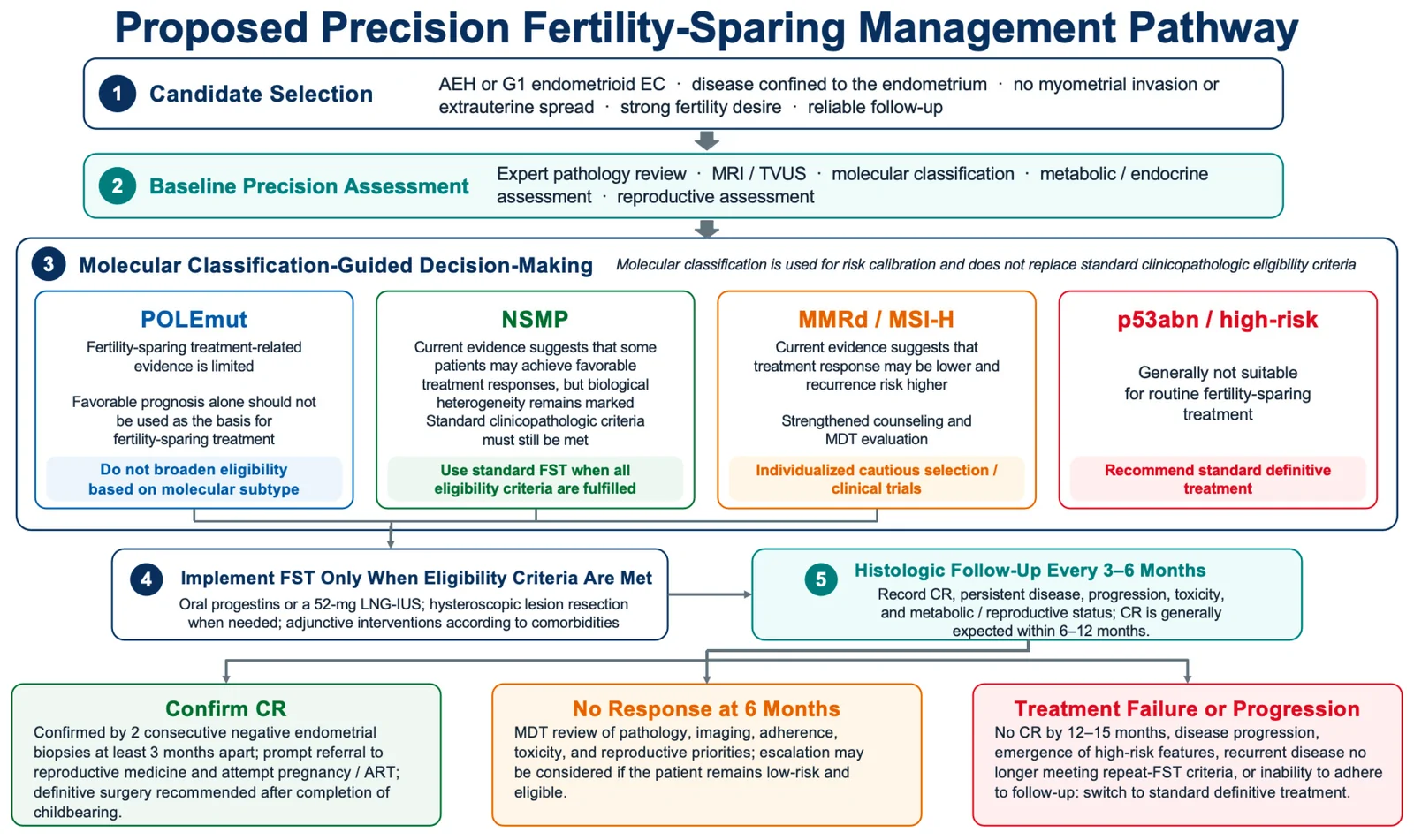
Proposed precision fertility-sparing management pathway. Abbreviations: AEH, atypical endometrial hyperplasia; ART, assisted reproductive technology; CR, complete response; EC, endometrial cancer; FST, fertility-sparing treatment; G1, grade 1; LNG-IUS, levonorgestrel-releasing intrauterine system; MDT, multidisciplinary team; MMRd, mismatch repair deficiency; MRI, magnetic resonance imaging; MSI-H, microsatellite instability-high; NSMP, no specific molecular profile; p53abn, p53-abnormal; POLEmut, POLE-mutated; TVUS, transvaginal ultrasonography.

The integrated clinical decision-making pathway is illustrated in Figure 1.

## 5. Limitations, Translational Challenges, and Unresolved Questions

The current evidence base for fertility-sparing treatment remains subject to important limitations. Existing studies show substantial heterogeneity in patient selection, histologic definitions, treatment regimens, surveillance schedules, and outcome reporting. Evidence for emerging strategies is derived largely from retrospective cohorts, small prospective studies, and case reports rather than randomized trials. A 2025 Cochrane review concluded that the overall certainty of evidence comparing different treatment strategies remains limited and that the available randomized evidence is insufficient to determine with confidence the optimal intervention, route of administration, or progestin dose [7]. Reproductive outcomes and the long-term safety of offspring are reported far less consistently than oncologic outcomes, and follow-up durations are generally insufficient to adequately assess late recurrence and long-term oncologic outcomes. These limitations may introduce bias into the existing literature and potentially lead to overestimation of the efficacy of some treatment strategies; therefore, encouraging findings from early-stage studies require further validation. Because this narrative review synthesizes evidence from these heterogeneous sources, its conclusions are likewise constrained by the methodological quality of the underlying studies and the maturity of the available evidence.

Real-world implementation also imposes important practical burdens. Long-term surveillance requires patients to adhere to repeated endometrial sampling, which may result in procedural discomfort, anxiety, time burden, and cumulative financial costs. Delaying definitive surgery inevitably exposes patients to a period of ongoing oncologic risk, and the balance between preserving reproductive opportunities and avoiding the risks associated with delayed definitive treatment may vary across individual patients and healthcare systems. Although international recommendations are broadly consistent regarding the selection of low-risk candidates, differences remain in the details of assessment and management. Access to expert pathologic review, molecular testing, hysteroscopy, reproductive medicine, and long-term multidisciplinary care is also uneven. These factors should be explicitly incorporated into shared decision-making rather than treated as secondary considerations. At present, evidence regarding the economic burden of fertility-sparing treatment remains limited, particularly when the combined costs of repeated biopsies, molecular testing, metabolic interventions, assisted reproductive technology, and delayed surgery are considered.

Translating precision fertility-sparing management into routine clinical practice will require standardized molecular testing and reporting, prospective validation of biomarkers predictive of progestin response and recurrence, harmonized definitions of complete response, resistance, and recurrence, and adequately powered studies incorporating pregnancy and live-birth outcomes. Artificial intelligence (AI), radiomics, and digital pathology represent promising adjunctive tools, but they cannot currently replace expert pathologic assessment or established molecular testing. A 2025 retrospective study including 102 patients undergoing fertility-sparing treatment evaluated an MRI radiomics–clinical model for predicting treatment response at 6 months and reported areas under the receiver operating characteristic curve (AUCs) of 0.941 and 0.907 in the training and test cohorts, respectively [42]. Another multicenter deep-learning digital pathology study demonstrated the feasibility of AI-based molecular classification of endometrial cancer using whole-slide images [43]. However, these tools are not yet ready for direct use in clinical candidate selection for fertility-sparing treatment. Before routine implementation, they require cross-institutional standardization, prospective clinical validation, assessment of model interpretability, and dedicated validation in fertility-sparing populations.

## 6. Conclusions and Future Research Priorities: From “Can Fertility Be Preserved?” to “How Can Precision Fertility-Sparing Management Be Achieved?”

Fertility-sparing treatment for endometrial cancer has evolved from oral progestin monotherapy into an integrated therapeutic framework encompassing the LNG-IUS, hysteroscopic lesion resection, metabolic interventions, combined endocrine therapy, molecular classification, and selected investigational systemic strategies. The value of this evolution lies not simply in expanding the number of available treatment options, but in enabling treatment intensity and mechanism of action to be better matched to each patient’s clinicopathologic risk, metabolic and endocrine characteristics, molecular subtype, treatment response, and reproductive goals. Established progestin-based strategies remain the clinical foundation of fertility-sparing treatment, whereas emerging metabolic, immunotherapeutic, and ER-targeted approaches should be interpreted according to the maturity and strength of the supporting evidence and should not be adopted beyond what the current data justify.

Future research priorities should include the following: (1) harmonizing definitions of complete response, inadequate response, progestin resistance, progression, and recurrence, and establishing core outcome sets encompassing both oncologic and reproductive outcomes; (2) prospectively validating molecular classifiers and biomarker combinations for patient selection and prediction of treatment response or recurrence; and (3) further defining the key mechanistic features of progestin resistance through integration of endocrine, metabolic, immune, and multi-omics data. Randomized controlled trials or pragmatic prospective studies should evaluate metabolic and combination strategies, including GLP-1-based approaches, with adequate follow-up for both recurrence and live-birth outcomes. Prospective studies should also clarify ovarian function, the optimal timing of conception, pregnancy outcomes, and offspring safety after immune checkpoint inhibitor or ER-targeted therapy. Artificial intelligence, radiomics, and digital pathology require further external validation, together with clearer evidence of their added value in clinical decision-making. The optimal timing of assisted reproductive technology (ART), the role of maintenance therapy, and retreatment strategies after recurrence also remain uncertain. Ultimately, the central principle of precision fertility-sparing management is to match each patient with the most appropriate intervention on the basis of the best available evidence, while clearly recognizing when oncologic safety must take precedence over uterine preservation.

## Figures and Tables

**Table 2:** Clinical trials of metabolic interventions in fertility-sparing treatment for endometrial cancer.

Study Focus	Registration Number	Status/Estimated Completion	Phase	Study Population	Intervention	Primary Endpoint/Fertility Outcomes
Tirzepatide + LNG-IUS	NCT07349641	Not yet recruiting; primary completion December 2028; study completion December 2029	Phase II	Overweight/obesity with AH/EIN or FIGO G1 endometrioid EC	Weekly tirzepatide + LNG-IUS	Pathologic CR at 26 weeks; sustained CR at 52 weeks; no dedicated pregnancy/live-birth endpoint
Tirzepatide + LNG-IUS (WE-FiERCE)	NCT06073184	Not yet recruiting; primary completion February 2027; study completion February 2032	Phase II	BMI ≥ 27; AH or low-grade EC; fertility preservation	Tirzepatide 2.5 mg weekly, escalated to 15 mg or the maximum tolerated dose + 52 mg LNG-IUS	Pathologic CR at 48 weeks; pregnancy, live birth, miscarriage, and pregnancy complications as secondary endpoints
GLP-1RA + LNG-IUS	NCT07107334	Recruiting; primary completion November 2028; study completion November 2029	Phase II	BMI ≥ 30; EIN or G1 endometrioid EC; desire for fertility preservation or medically inoperable	Clinician-selected GLP-1RA + LNG-IUS	Pregnancy attempts or surgery permitted; pathologic response at 6/12 months; no dedicated pregnancy/live-birth endpoint
Behavioral weight loss + LNG-IUS (UPLifT-Endo)	NCT05903131	Recruiting; primary completion October 2028; study completion October 2029	Phase II	Premenopausal obesity with AEH/G1 EC; uterine-preserving management	Behavioral weight loss + LNG-IUS vs. LNG-IUS alone	No AEH on biopsy at 1 year; no dedicated pregnancy/live-birth endpoint

Abbreviations: AEH, atypical endometrial hyperplasia; AH, atypical hyperplasia; BMI, body mass index; CR, complete response; EC, endometrial cancer; EIN, endometrial intraepithelial neoplasia; FIGO, International Federation of Gynecology and Obstetrics; G1, grade 1; GLP-1RA, glucagon-like peptide-1 receptor agonist; LNG-IUS, levonorgestrel-releasing intrauterine system. Trial information and recruitment status were obtained from ClinicalTrials.gov (accessed on 10 August 2026).

**Table 3:** Clinical trials of immunotherapy and molecular classification in fertility-sparing treatment for endometrial cancer.

Study Focus	Registration Number	Status/Estimated Completion	Phase	Study Population	Intervention	Primary Endpoint/Fertility Outcomes
PD-1 inhibitor + progestin	NCT06549855	Not yet recruiting; primary completion July 2029; study completion October 2029	Not applicable	Early-stage MMRd endometrioid EC; fertility preservation	Sintilimab or pembrolizumab 200 mg intravenously every 3 weeks + MA 320 mg/day or MPA 500 mg/day	Complete response rate at approximately 3 months; no dedicated pregnancy/live-birth endpoint
TQB2450 + progestin	NCT06914297	Recruiting; primary completion October 2027; study completion April 2029	Phase II	MMRd EC, FIGO 2023 IA1–IA2	TQB2450 1200 mg intravenously every 3 weeks + MA 160 mg/day or MPA 500 mg/day	3-month CR rate (12–16 weeks); pregnancy and live birth as secondary endpoints

Abbreviations: CR, complete response; EC, endometrial cancer; FIGO, International Federation of Gynecology and Obstetrics; MA, megestrol acetate; MMRd, mismatch repair deficiency; MPA, medroxyprogesterone acetate; PD-1, programmed cell death protein 1. Trial information and recruitment status were obtained from ClinicalTrials.gov (accessed on 10 August 2026).

## Data Availability

No new data were created or analyzed in this study. Data sharing is not applicable to this article.

## Abbreviations

**Abbreviation**	**Full term**
EC	Endometrial cancer
FST	Fertility-sparing treatment
G1	Grade 1
AEH	Atypical endometrial hyperplasia
FIGO	International Federation of Gynecology and Obstetrics
ESGO	European Society of Gynaecological Oncology
ESHRE	European Society of Human Reproduction and Embryology
ESGE	European Society for Gynaecological Endoscopy
NCCN	National Comprehensive Cancer Network
ACOG	American College of Obstetricians and Gynecologists
MRI	Magnetic resonance imaging
LNG-IUS	Levonorgestrel-releasing intrauterine system
MPA	Medroxyprogesterone acetate
MA	Megestrol acetate
PCOS	Polycystic ovary syndrome
GLP-1	Glucagon-like peptide-1
GLP-1RA	Glucagon-like peptide-1 receptor agonist
GIP	Glucose-dependent insulinotropic polypeptide
BMI	Body mass index
MMRd	Mismatch repair deficiency
MSI-H	Microsatellite instability-high
POLEmut	POLE exonuclease domain mutation
NSMP	No specific molecular profile
AH	Atypical hyperplasia
EIN	Endometrial intraepithelial neoplasia
GnRH-a	Gonadotropin-releasing hormone agonist
IGF-1	Insulin-like growth factor-1
ER	Estrogen receptor
PR	Progesterone receptor
PGR	Progesterone receptor gene
PI3K	Phosphoinositide 3-kinase
AKT	Protein kinase B
AMPK	AMP-activated protein kinase
mTOR	Mechanistic target of rapamycin
MAPK	Mitogen-activated protein kinase
IL-6	Interleukin-6
TNF-α	Tumor necrosis factor-α
NF-κB	Nuclear factor κB
STAT3	Signal transducer and activator of transcription 3
DHCR24	24-Dehydrocholesterol reductase
MMR	Mismatch repair
PD-1	Programmed cell death protein 1
PD-L1	Programmed death-ligand 1
CR	Complete response
ART	Assisted reproductive technology
MDT	Multidisciplinary team
RFS	Recurrence-free survival
AI	Artificial intelligence
AUC	Area under the receiver operating characteristic curve
